# Distinct synaptic and neurochemical changes to the granule cell-CA3 projection in Bassoon mutant mice

**DOI:** 10.3389/fnsyn.2015.00018

**Published:** 2015-10-23

**Authors:** Sandra Dieni, Sigrun Nestel, Mirjam Sibbe, Michael Frotscher, Sabine Hellwig

**Affiliations:** ^1^Neurochemistry Laboratory, Department of Molecular Psychiatry, University Hospital FreiburgFreiburg, Germany; ^2^Department of Neuroanatomy, Institute of Anatomy and Cell Biology, University of FreiburgFreiburg, Germany; ^3^Institute for Structural Neurobiology, Center for Molecular Neurobiology HamburgHamburg, Germany

**Keywords:** synaptic plasticity, hippocampal ultrastructure, mossy fiber bouton, dense-core vesicle, microglia

## Abstract

Proper synaptic function depends on a finely-tuned balance between events such as protein synthesis and structural organization. In particular, the functional loss of just one synaptic-related protein can have a profound impact on overall neuronal network function. To this end, we used a mutant mouse model harboring a mutated form of the presynaptic scaffolding protein Bassoon (*Bsn*), which is phenotypically characterized by: (i) spontaneous generalized epileptic seizure activity, representing a chronically-imbalanced neuronal network; and (ii) a dramatic increase in hippocampal brain-derived neurotrophic factor (BDNF) protein concentration, a key player in synaptic plasticity. Detailed morphological and neurochemical analyses revealed that the increased BDNF levels are associated with: (i) modified neuropeptide distribution; (ii) perturbed expression of selected markers of synaptic activation or plasticity; (iii) subtle changes to microglial structure; and (iv) morphological alterations to the mossy fiber (MF) synapse. These findings emphasize the important contribution of Bassoon protein to normal hippocampal function, and further characterize the *Bsn*-mutant as a useful model for studying the effects of chronic changes to network activity.

## Introduction

Proper hippocampal function depends on a finely-tuned balance between excitation and inhibition within the neuronal network, where any changes to this setting can have secondary effects on events such as protein synthesis and structural organization. A case in point is a genetic mouse model that is deficient in the full-length form (420 kD) of the presynaptic scaffolding protein Bassoon, but instead expresses a shorter form of the protein (180 kD) lacking the regions encoded by exons 4 and 5 of the Bassoon (*Bsn*) gene (Altrock et al., 2003). It is assumed that many functions of *Bsn* are abrogated in this mutant, with major effects on synaptic transmission that manifest phenotypically as episodic generalized seizures in mature mutant mice (Altrock et al., 2003; Ghiglieri et al., 2009). This renders the *Bsn*-mutant mouse model as a means by which to monitor the effects of perturbed neuronal network activity.

In addition to the changes in synaptic function, macroscopic analyses of the *Bsn*-mutant brain have demonstrated functional and structural alterations to cortical structures. Manganese-enhanced magnetic resonance (MR) imaging and metabolic labeling with [14C]-2D-deoxyglucose revealed altered cortical activation patterns in *Bsn*-mutant mice (Angenstein et al., 2007), while a follow-up MR spectroscopy study uncovered a reduction in neuronal density in cortical layer V but found an overall increase in cortical and hippocampal volumes (Angenstein et al., 2008). A more detailed analysis of the *Bsn*-mutant hippocampus at the microscopic level confirmed that hippocampal volume is enlarged, with stereological quantification of principal neurons in the dentate gyrus (DG) and Ammon’s horn revealing enhanced neurogenesis and reduced apoptosis (Heyden et al., 2011). Changes in certain aspects of hippocampal plasticity were identified as potential correlates of the epileptic seizure activity in *Bsn*-mutant mice, where CA1 neurons were found to harbor abnormal dendrite structure and failed to elicit long term potentiation (LTP; Sgobio et al., 2010). Furthermore, mossy fiber boutons (MFBs), which form a major synapse with CA3 complex spines and play an important role in hippocampal plasticity (Evstratova and Tóth, 2014), were found to undergo an abnormal course of physiological maturation during the postnatal period (Lanore et al., 2010).

One of the most striking neurochemical changes identified at the subcellular level in the *Bsn*-mutant brain thus far is the dramatic increase in hippocampal brain-derived neurotrophic factor (BDNF) protein concentration (Heyden et al., 2011). This was subsequently confirmed at the immunohistochemical level, where intense BDNF immunoreactivity was confined to hippocampal neuropil layers and largely corresponded to increased presynaptic BDNF storage in MFBs (Dieni et al., 2012).

Given the well-defined role of BDNF in the regulation of synaptic plasticity and transmission in the adult brain (reviewed in Park and Poo, 2013), the present study investigated whether other mechanisms known to modulate certain aspects of synaptic plasticity were also affected by the altered physiological environment that manifests in the *Bsn*-mutant hippocampus. Immunohistochemical and morphometric analyses of hippocampi at the light and electron microscopic level revealed that the increased presynaptic levels of BDNF in *Bsn*-mutants are accompanied by changes in the expression of other key neurochemical and structural markers of neuronal plasticity.

## Materials and Methods

### Mouse Lines

Brain tissue was derived from *Bsn*-mutant mice, a mouse line lacking the full-length (420 kD) form of Bassoon (*BsnΔEX4*/5; see Altrock et al., 2003). Wild-type (WT) littermates were used as controls. All experiments were performed in accordance with the institutional guidelines of the University of Freiburg. The use of animals was approved by the Animal Welfare Ethics Committee from the Regional Council of Freiburg.

### Preparation of Tissue for Immunostaining

Eight-week-old mice (*n* = 7 per genotype) were transcardially perfused with 0.9% NaCl followed by a mixture of 4% PFA and 0.1% glutaraldehyde in 0.1 M phosphate buffer (PB), pH 7.4. Brains were removed and post-fixed with 4% PFA in 0.1 M PB for 1 h before thorough washing in 0.1 M PB. Coronal sections (50 μm) were cut along the septotemporal axis of the hippocampus on a vibratome (Leica) and further processed for either immunohistochemistry or pre-embedding immunogold labeling.

### Antibodies for Immunostaining

BDNF was detected with the mouse monoclonal BDNF antibody Mab#9 (anti-BDNF; Kolbeck et al., 1999) directed against the mature domain of BDNF (Dieni et al., 2012). For postsynaptic labeling, a rabbit polyclonal antibody against Synaptopodin (“anti-synpo”; #163002; Synaptic Systems), and a guinea pig polyclonal antibody against activity-related cytoskeletal protein (“anti-Arc”; #156005; Synaptic Systems) were used. For labeling of neuropeptides within the MF projection, rabbit polyclonal antibodies against Met-enkephalin (“anti–Met-enk”; Millipore) and Cholecystokinin (anti-CCK; #P06307; Millipore) were applied. Microglia were detected with a rabbit polyclonal antibody against ionized calcium-binding adaptor molecule 1 (“anti-Iba1”; #019-19741; Wako Chemicals). Reelin was detected with a mouse monoclonal Reelin (clone G10) antibody (“anti-Reelin”; #MAB5364; Millipore), while for Calbindin-D28K (CB) labeling, a polyclonal rabbit antibody (“anti-CB”; #CB-38a; Swant) was used. 4^′^6-Diamidine-2-phenylindol (DAPI, 1:1000, 1 mg/mL solution, ThermoScientific) was used for nuclear counterstaining.

### Immunofluorescence

Antigen retrieval (30 min in sodium citrate buffer (pH 8.5) at 80°C; Jiao et al., 1999) was performed on WT and *Bsn*-mutant sections prior to antibody application. The staining protocol for all antibodies was identical, and 50 mM tris-buffered saline (TBS, pH 7.4) was used throughout to wash the sections. Sections were blocked either with 3% M.O.M. (Mouse-on-Mouse blocking serum, Vector Laboratories; for anti-BDNF only) or 20% normal donkey serum (NDS) for 1 h at RT. Primary antibodies were diluted in a solution of 3% bovine serum albumin (BSA), 2% NDS, and 0.2% Triton X-100 in TBS to yield the following final concentrations/dilutions: 10 μg/ml anti-BDNF, 2 μg/ml anti-Arc, 1:000 anti-synaptopodin, 3 μg/ml anti–Met-enk, 1:2000 anti-CCK, 1:1000 anti-Iba1, 1:250 anti-Reelin, 1:1000 anti-Calbindin and 1:1000 anti-Iba1. Sections were incubated for 1–3 nights at 4°C in 1–2 primary antibodies. For fluorescent signal detection, the following Alexa Fluor–conjugated secondary antibodies were used: donkey anti–rabbit-488 or -555, donkey anti–mouse-555 or -647, and donkey anti–guinea pig–Cy5 (1:400; all purchased from Millipore, Germany). Labeled sections were mounted onto glass slides and coverslipped with fluorescent mounting medium (Dako).

### Confocal Microscopy

Immuno-labeled hippocampal sections were viewed on a Zeiss Axiovert 200 inverted microscope equipped with Plan-Apochromat 20× (numerical aperture, NA: 0.75) or 63× (oil DIC; NA: 1.4) objectives and attached to a spectral confocal laser system (LSM 510, Carl Zeiss) powered by Zeiss LSM 4.2 Meta Software (Carl Zeiss Microimaging, Germany). The tissue was scanned with 488 nm, 543 nm and 633 nm laser lines to detect the corresponding Alexa fluorophores. High resolution images (1024 × 1024 pixels) of optical sections (z-slices) were captured using sequential line (average of 4) scanning. Maximal projection images of confocal z-series (stacks) were generated where indicated in the figure legends. Images were arranged and annotated using Corel Draw 12 software (Corel Corporation).

### Quantification and Data Analyses of Immunohistochemistry

#### Cell Counts

The density of Reelin- or Arc-labeled cells was determined in three coronal sections of septal hippocampus per animal (*n* = 3–4 animals per genotype). Cell numbers were estimated bilaterally in the DG, and the area of interest was measured using ImageJ 1.40 analysis software (National Institutes of Health). Cell densities are expressed as the number of cells per 100 μm^2^.

#### Quantitative Assessment of Met-Enk-, CCK- and Calbindin-Staining Intensity (SI)

Single plane confocal images of each immunohistochemical marker were converted into a black and white binary format in ImageJ, and the relative numbers of black and white pixels within a defined region of interest (ROI) of the MF pathway (i.e., hilus and stratum lucidum, SL) were automatically generated. For each immunohistochemical marker, the ROI area in which the pixels were counted was identical in each section measured. The number of white pixels were deemed proportional to the fluorescence SI, whereby sections with increased immunostaining had a higher fraction of white pixels. The mean percentage of white pixels was calculated for each marker per group.

#### Assessment of Synaptopodin-SI

Two investigators (SD, SH) blinded to genotype data independently assessed synaptopodin staining intensity (SI) in the entire molecular layer (ML) of the DG, as well as in the inner (IML), middle (MML) and outer (OML) sublayers (*n* = at least 3–4 animals per genotype) according to an *a priori* defined score (0 = no staining, 1 = weak SI, 2 = moderate SI, 3 = strong SI). In cases of discrepant ratings, a consensus between both investigators was reached.

#### Three-Dimensional (3D) Reconstruction of Microglia

Confocal z-scans of hilar Iba-1 positive microglia were acquired through a 40× objective (NA 1.4, oil immersion) at 0.8 μm intervals. Image series were saved in lsm format and opened in NeuronStudio software (Wearne et al., 2005). Each cell (*n* = 15–20 per genotype) was manually traced using the neurite tool and a 3D model was generated thereafter. An automated Sholl analysis was also performed on each digitized cell.

### BDNF Immunogold Electron Microscopy

Fifty micro meter vibratome sections from WT and *Bsn*-mutants were processed for pre-embedding immunogold labeling as previously described (Dieni et al., 2012). Briefly, sections were snap frozen to increase antigenicity, blocked in 3% M.O.M. and incubated in anti-BDNF (20 μg/ml) for three nights at 4°C. After thorough washing with 50 mM TBS, sections were treated overnight at 4°C with 1.4 nm gold-conjugated mouse IgG (1:100). Bound gold particles were enhanced using a silver intensification kit (HQ Silver; Nanoprobes) and sections were then fixed for 10 min in 1% GA solution. Sections underwent osmification (0.5% OsO_4_ and 6.86% sucrose in 0.1 M PB) for 40 min, were treated with 1% uranyl acetate in 70% ethanol (EtOH) for 35 min, and dehydrated in increasing grades of EtOH. After washing in propylene oxide, the tissue was embedded in Durcupan (Fluka). Ultrathin sections (60 nm) of CA3 were cut and mounted on formvar-coated copper grids.

### Morphometric Analyses of Mossy Fiber Boutons

#### Mossy Fiber Bouton/CA3 Spine Dimensions

For measurement of MFB profile areas and folding indices, as well as relative spine areas and numbers per bouton, the SL was located in ultrathin sections mounted onto a LEO-906E electron microscope (Zeiss) and optically viewed at a magnification of 6000. Composite digital images (3 × 3) were then captured and assembled using a TRS “sharp-eye” HSC 2048 digital camera system (Schneider Systemtechnik GmbH, Germany) and the “Multiple Image Acquisition” function in AnalySIS acquisition software (Soft Imaging Systems, Olympus, Germany). Captured images from WT (*n* = 5) and *Bsn-*mutant (*n* = 7) sections were opened in the analysis software Cell^∧^P (Soft Imaging Systems, Olympus, Germany) and MFBs were unambiguously identified according to their well-defined morphology (i.e., high density of synaptic vesicles, numerous synaptic contacts with CA3 complex spines, non-synaptic puncta adherentia at dendritic shafts, relatively large surface area). The perimeter and area of each identified MFB and its associated spines were measured. The folding index of each MFB was then calculated by dividing the area by the perimeter, whereby a relative lower ratio represented a higher degree of folding (Zhao et al., 2012). At least 20 MFBs and 60 spines per animal (WT: *n* = 5; Bsn: *n* = 7) were analyzed.

#### Dense Core Vesicles Distance from Presynaptic Membrane

MFBs containing labeled dense core vesicles (DCV) were then selected for analyses, in which the direct distance to the membrane of each DCV was determined with the straight line measuring tool in Image J. Seventeen BDNF-labeled MFBs in WT mice (*n* = 5), and 12 labeled MFBs in *Bsn*-mutants (*n* = 3) were analyzed.

### Data Acquisition and Statistical Analysis

Mean values per animal were calculated for each parameter analyzed and group means (WT vs. *Bsn*-mutant) were compared. The software package SPSS19^1^ was used for statistical analyses. Statistical differences between groups were assessed using a Student’s *t*-test (normally-distributed data), or a non-parametric Wilcoxon-test (two-sided). To test for the effects of genotype and distance from soma on microglial morphology, a two-factorial analysis of variance (ANOVA) with *post hoc* Tukey-Kramer HSD test was applied. In all tests, significance was assigned when *p* < 0.05. All values are expressed as mean ± SEM.

## Results

### Met-Enkephalin and Cholecystokinin show Differential Changes in Immunohistochemical Distribution in *Bsn*-Mutants

**Figure 1:**
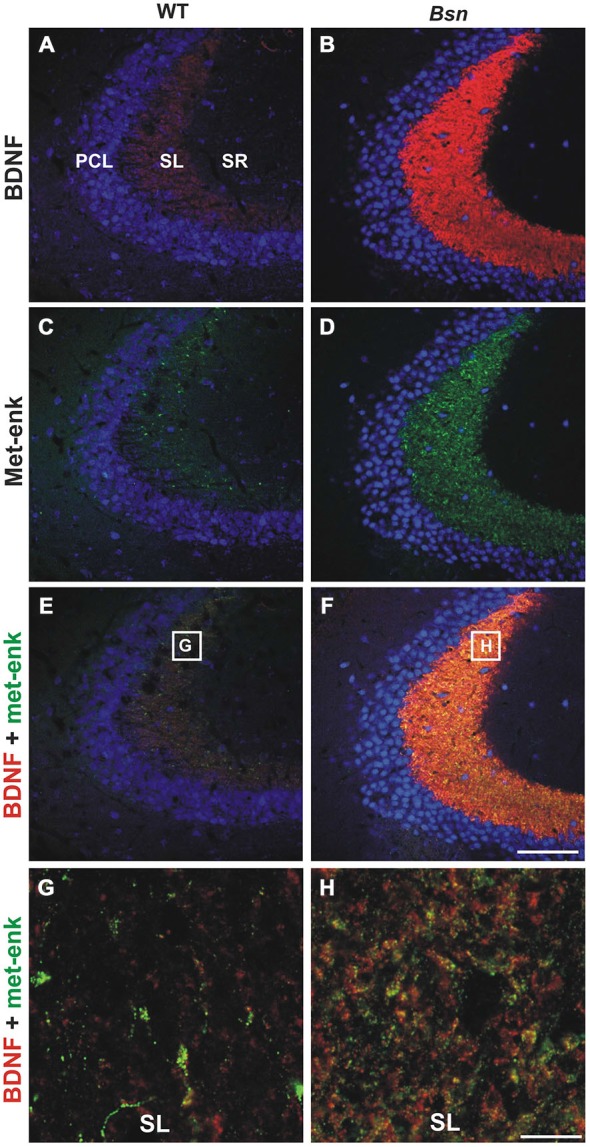
**Met-enkephalin-IR and brain-derived neurotrophic factor (BDNF) are upregulated in parallel in the hippocampus of Bassoon mutants.** BDNF (red)/met-enk (green) co-labeling in coronal hippocampal sections (CA3) from adult wild-type (WT) mice **(A,C,E)** and *Bsn*-mutants **(B,D,F)**. Note the concurrent upregulation of **(B)** BDNF and **(D)** Met-enk in SL of *Bsn*-mutants compared to WT **(A,C)**. High resolution view of SL reveals an increased proportion of BDNF- and Met-enk-double-labeled MFBs in **(H)** *Bsn*-mutant compared to **(G)** WT mice. Nuclei are labeled with DAPI (blue). Scale bars: **A–F**: 100 μm; **G,H**: 10 μm. Abbreviations: PCL, pyramidal cell layer of CA3; SL, stratum lucidum; SR, stratum radiatum.

Based on the observation that *Bsn*-mutants display a dramatic increase in hippocampal BDNF protein concentration (Heyden et al., 2011), particularly in the MF projection pathway (Dieni et al., 2012), it was of interest to investigate whether the immunohistochemical expression of two other small neuropeptides that are normally localized to MFs and known to modulate certain aspects of neuronal excitability was also affected in the *Bsn*-mutant. Hippocampal sections were first stained with antibodies against the methionine form of enkephalin (Met-enk), a small opioid that is derived from pro-enkephalin and stored in DCVs (Cheng et al., 1995). In WT hippocampi, Met-enk immunoreactivity (-IR; Figure 1C) showed a similar distribution pattern to BDNF (Figure 1A) in SL, although it appeared to be confined to a smaller subset of MFBs (Figures 1C,E,G). In *Bsn*-mutant mice, Met-enk-IR showed an upregulation in SL (Figure 1D), similar to BDNF-IR (Figure 1B). This difference was confirmed by quantifying the immunofluorescent signal in terms of pixel intensity in the MF pathway (proportion of white pixels in WT: 34.7 ± 4.4% vs. Bsn: 74.7 ± 3.1%; 2-sided *t*-test: *p* < 0.0001). Image overlay revealed a higher degree of overlap in comparison to WT sections between Met-enk-positive (+) and BDNF+ puncta in SL (Figure 1F), which at higher resolution was shown to correspond to a greater proportion of BDNF+/Met-enk+ MFB profiles (Figure 1H). Therefore, Met-enk shows a strong increase in SI in parallel with BDNF, thus leading to a putative increase in the number of MFBs that contain both peptides. This suggests that BDNF and Met-enk peptides might respond to similar stimuli for their transcription and release.

**Figure 2:**
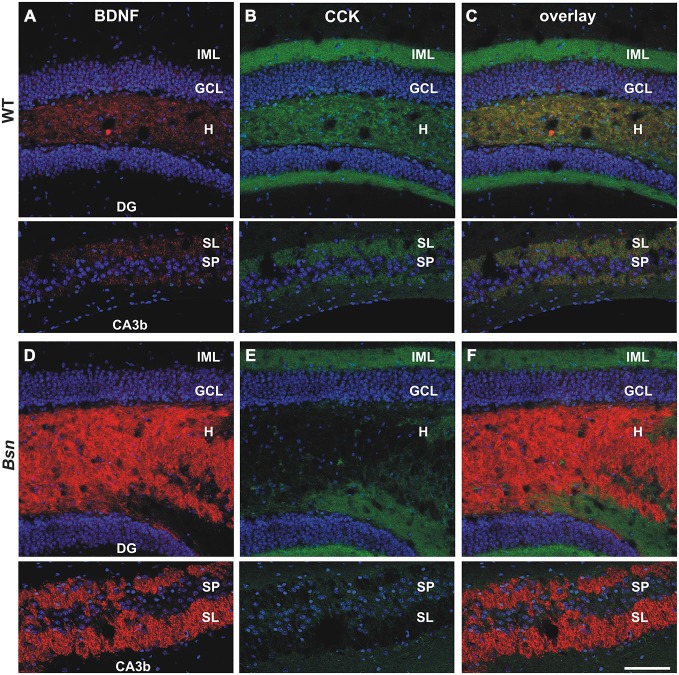
**Selective loss of CCK-IR in the mossy fiber (MF) projection pathway of Bassoon mutants.** Co-labeling with antibodies against BDNF (red) and CCK (green) revealed a selective loss of CCK-IR in the MF projection pathway of *Bsn*-mutants. **(A)** BDNF and **(B)** CCK-IR in the dentate gyrus (DG) (upper images) and CA3b (lower images) regions of an adult WT mouse. **(C)** Merged image of BDNF/CCK-IR reveals segregation of the two neuropeptide signals throughout the DG and CA3. Whereas the intensity of **(D)** BDNF-IR is elevated in the *Bsn*-mutant hilus and SL, there is a dramatic loss of **(E)** CCK-IR in granule cell axon terminals (i.e., MFBs) in the hilus and SL. **(F)** Merged image of BDNF/CCK-IR emphasizes upregulation of BDNF-IR, and corresponding down-regulation of CCK-IR in the hilus and SL of *Bsn*-mutants. Nuclei are labeled with DAPI (blue). Scale bar: 100 μm. Abbreviations: IML, inner molecular layer; GCL, granule cell layer; H, hilus; DG, dentate gyrus; SL, Stratum Lucidum; SP, Stratum Pyramidale.

Confocal scanning was next performed on hippocampal sections labeled with antibodies against cholecystokinin (CCK), a hormone peptide that is not only expressed in hippocampal interneurons but also transported along the MF pathway of the ventral hippocampus in mice (Gall et al., 1986). CCK is localized to a subset of MFB profiles (Figure 2B) but is not co-expressed with BDNF protein (Figures 2A,C; see also Dieni et al., 2012). In contrast to BDNF (Figure 2D) and Met-enk (Figure 1D), a clear selective loss of CCK-IR was observed in the hilus (Figures 2E,F, upper images) and SL (Figures 2E,F, lower images) of *Bsn*-mutants compared to control mice (Figures 2B,C). Quantification of SI in MFs confirmed the downregulation of CCK-IR in *Bsn*-mutants (proportion of white pixels in WT: 65.5 ± 4.0% vs. Bsn: 4.2 ± 1.7%; 2-sided *t*-test: *p* < 0.0001). Therefore, CCK appears to be depleted from *Bsn* MFBs, despite the elevated levels of BDNF and Met-enk.

The neuropeptide staining data therefore suggest that not all small neuropeptides are governed by the same regulation mechanisms, and that the normal relative proportions of neuropeptides are perturbed in *Bsn*-mutants.

### Calbindin Expression is Reduced in the Hippocampus of *Bsn*-Mutants

**Figure 3:**
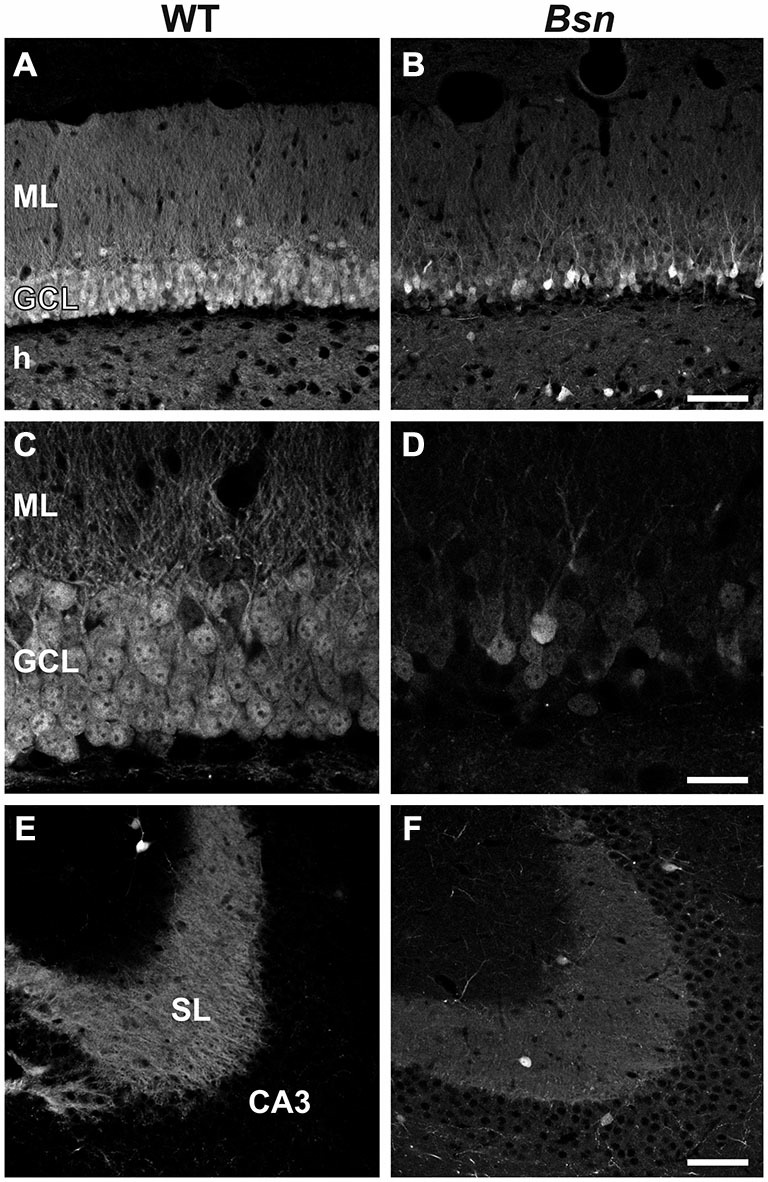
**Loss of Calbindin immunoreactivity in *Bsn*-mutant dentate granule cells. (A)** Normal distribution of CB-IR in the WT DG. Note uniform distribution of immunoreactivity throughout the granule cell bodies and their associated dendrites and MFs in the ML and hilus, respectively. **(B)** Loss of CB-IR in granule cells, dendrites and MFs in the *Bsn*-mutant DG. Note that the outer GCL is particularly affected by this loss. **(C,D)** Higher magnification view of the GCL and ML in WT vs. *Bsn*-mutants. **(E)** Strong, uniform CB-IR in MFs contained within SL of hippocampal area CA3. Comparison with CB-IR in the **(F)** *Bsn*-mutant reveals a downregulation in staining intensity (SI), indicating reduced CB protein content in MFs. Scale bars: **A,B,E,F** = 100 μm; **C,D** = 20 μm. Abbreviations: ML, molecular layer; GCL, granule cell layer; h, hilus; SL, stratum lucidum.

CB is a calcium-binding protein expressed in subsets of neurons including dentate granule cells, and serves as a useful marker of cell viability (Celio, 1990). Moreover, increased neuronal activity in *Bsn*-mutant mice is likely to be accompanied by increased NMDA-mediated calcium influxes (Fujikawa, 2005). Therefore, we compared CB-IR between WT animals and *Bsn*-mutants. *Bsn*-mutant mice (Figure 3B) displayed a marked reduction in granule cell CB-IR compared to WT animals (Figure 3A), particularly in the outer- and innermost bands of the layer (Figures 3C,D). This was accompanied by lower CB-IR within the MF projection pathway (proportion of white pixels in WT: 70.2 ± 6.9% vs. Bsn: 10.3 ± 3.6%; 2-sided *t*-test: *p* < 0.0001; Figures 3E,F). The marked loss of CB-IR in the DG of *Bsn*-mutants therefore points to a potential compromise in granule cell calcium homeostasis and ultimately cell function.

### Alterations in the Expression Patterns of Synaptic Activation Markers

**Figure 4:**
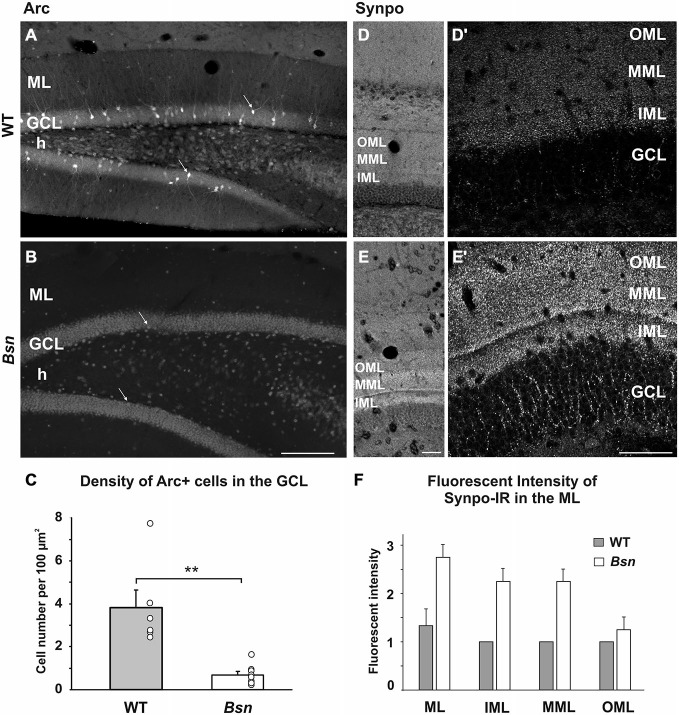
**Differential effects of neuronal network hyperexcitability on markers of synaptic plasticity. (A–C)** Density of Arc+ cells in the DG. **(A)** Varying degrees of Arc-staining intensity were found in a subset of granule cells (arrows) in the WT DG. **(B)** Only a few Arc+ granule cells were observed in the *Bsn-*mutant. **(C)** Quantification of Arc+ cells in the DG revealed a significant reduction in *Bsn-*mutants compared to WT mice. **(D–F)** SI of Synpo-IR in the DG. **(D)** Uniform distribution of Synpo+ puncta across the sublayers of the ML. **(D^′^)** Delineation between the IML and MML can be observed at higher magnification in WT mice. **(E)** Increased Synpo-IR intensity of the MML and IML in the *Bsn-*mutant DG. Note the even stronger delineation between the IML and MML in **(E^′^)**. Synpo staining in the GCL is also increased. **(F)** Grading of Synpo-IR intensity confirms a relative increase in fluorescence intensity in the inner two thirds of the ML in *Bsn*, while no change was observed between genotypes in the OML (Intensity scale: 0, no signal; 1, weak; 2, moderate; 3, strong signal). Data are shown as mean ± SEM. **p < 0.01. Scale bars: **A,B** = 200 μm; **D,D^′^,E,E^′^**: 100 μm. Abbreviations: ML, molecular layer; GCL, granule cell layer; h, hilus, Synpo, Synaptopodin; OML, outer molecular layer; MML, middle molecular layer; IML, inner molecular layer.

It is well established that BDNF transcription is activity-dependent and through its predominant presynaptic localization (Andreska et al., 2014), ultimately promotes synaptic plasticity (reviewed in Park and Poo, 2013). Given that BDNF levels are increased in the *Bsn*-mutants, it was important to determine whether this was associated with an upregulation of other markers normally influenced by elevated synaptic activity. Therefore, we first examined sections labeled with antibodies against Arc/Arg3.1, an immediate early gene (IEG) whose expression is increased in parallel with synaptic activity, whereby the mRNA product is locally translated into protein both in somata and dendrites (Lyford et al., 1995). In the DG of WT animals, Arc protein was distributed in a subset of granule cells, where the immunoreactivity extended from the cell body to the most distal parts of the dendrites (Figure 4A). Despite the dramatic increase in BDNF protein in *Bsn*-mutants, Arc expression was surprisingly sparse, with only scattered granule cells showing weak somal staining and minimal dendritic labeling (Figure 4B). Quantification of Arc+ cell density confirmed this reduction in staining in *Bsn*-mutants compared to WT animals (*Bsn*-mutant 0.68 ± 0.16 vs. WT: 3.83 ± 0.81 cells per 100 μm, *p* = 0.001; two-sided Student’s *t*-test; Figure 4C).

Synaptopodin is an actin-associated protein that is localized to the spine apparatus in excitatory neurons (reviewed in Deller et al., 2007) and distributed in a laminar pattern within the hippocampus (Bas Orth et al., 2005). In the DG of WT sections labeled with synaptopodin antibodies, immunoreactive puncta corresponding to labeled spines on granule cell dendrites were distributed throughout the entire ML, with a staining gradient ranging from stronger to weaker across the three sublayers of the ML (Figure 4D). A clear delineation between the inner (IML) and middle molecular layers (MML) was also observed (Figure 4D^′^; Bas Orth et al., 2005). In the *Bsn*-mutant DG, synaptopodin SI was markedly increased in comparison to WT sections (Figure 4E), with larger fluorescent puncta and an even stronger delineation between the IML and MML (Figure 4E^′^). Qualitative assessment of synaptopodin SI within the ML confirmed that synaptopodin-IR is markedly increased in this region of the *Bsn*-mutant hippocampus (Figure 4F). These data suggest that the chronic imbalance in neuronal activity in these mutants leads to the upregulation of a post-synaptic actin-associated protein, which might correspond to an increase in spine size or number in this region.

### Reelin Protein Distribution is Unchanged in the DG of *Bsn*-Mutants

**Figure 5:**
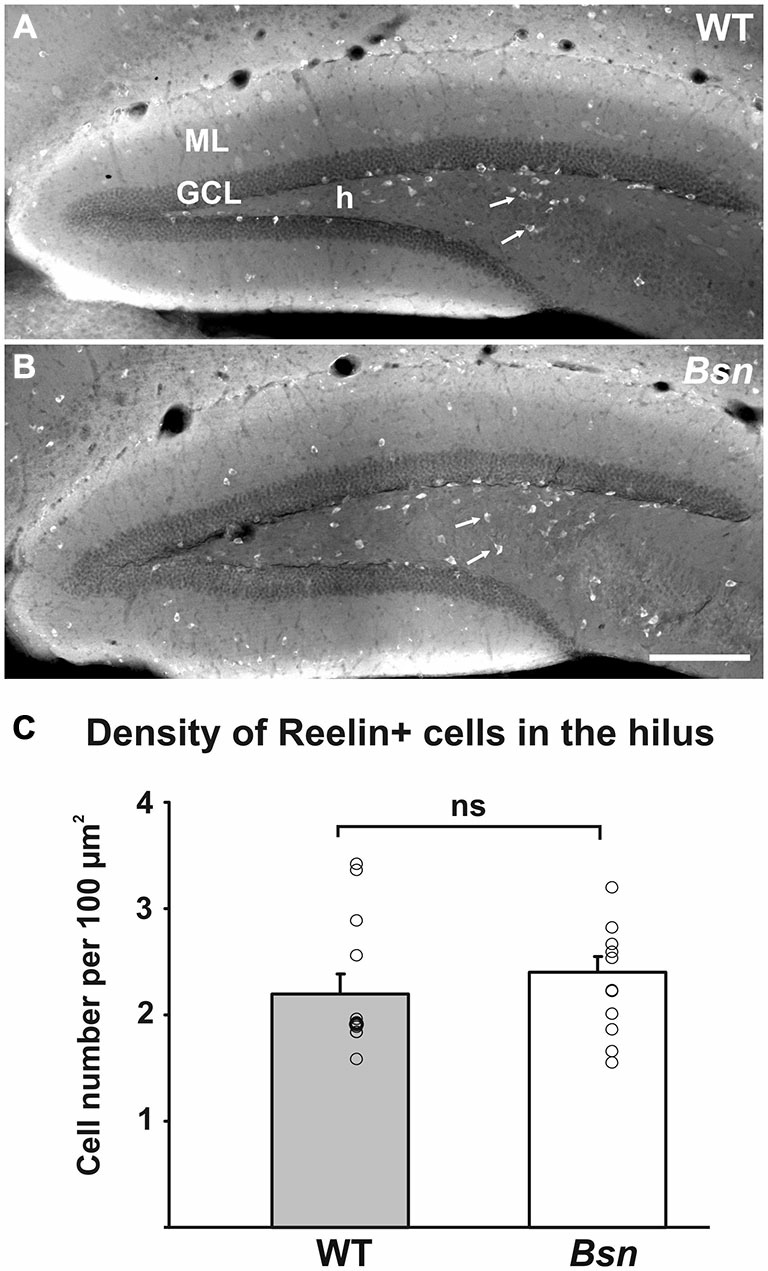
**Distribution of Reelin immunoreactivity in the DG is comparable between WT and *Bsn*-mutant mice.** Distribution of Reelin+ neurons (arrows) in the **(A)** WT and **(B)** *Bsn*-mutant DG. **(C)** Measurement of Reelin+ neuron density in the hilus revealed a lack of significant difference between genotypes. Data are shown as mean ± SEM. Circles represent single data points. Scale bar = 200 μm. Abbreviations: ML, molecular layer; GCL, granule cell layer; h, hilus; ns, not significant.

Reelin is an extracellular matrix protein well-characterized for its essential role in cortical lamination (Frotscher, 2010). In the mature brain, Reelin is synthesized by a subpopulation of GABAergic interneurons (Drakew et al., 1998; Pesold et al., 1998). Reelin continues to play a role beyond development and has been implicated in the modulation of neurotransmission (Hellwig et al., 2011), NMDA receptor activity (Chen et al., 2005) and neurogenesis (Sibbe et al., 2015), and may underlie the granule cell dispersion (GCD) that characterizes the DG in temporal lobe epilepsy (TLE; Haas et al., 2002). To determine whether Reelin expression by inhibitory GABAergic interneurons was affected in the present model of neuronal hyperactivity, the DGs of Reelin-labeled hippocampi were examined. In WT hippocampal sections, Reelin-IR was distributed across the bands of neuropil flanking the hippocampal fissure and was also strongly expressed by hilar GABAergic interneurons (Figure 5A). No qualitative differences in Reelin-IR in these neuropil layers were observed between WT and *Bsn*-mutant sections. To determine whether the distribution of Reelin+ hilar interneurons was affected in *Bsn*-mutants (Figure 5B), the number of labeled cells in the hilus was quantified and compared between WT and mutants. Here it was found that the density of Reelin+ cells remained unchanged in the *Bsn*-mutant (Figure 5C).

### Loss of Bassoon is Associated with Altered Microglial Morphology in the DG

**Figure 6:**
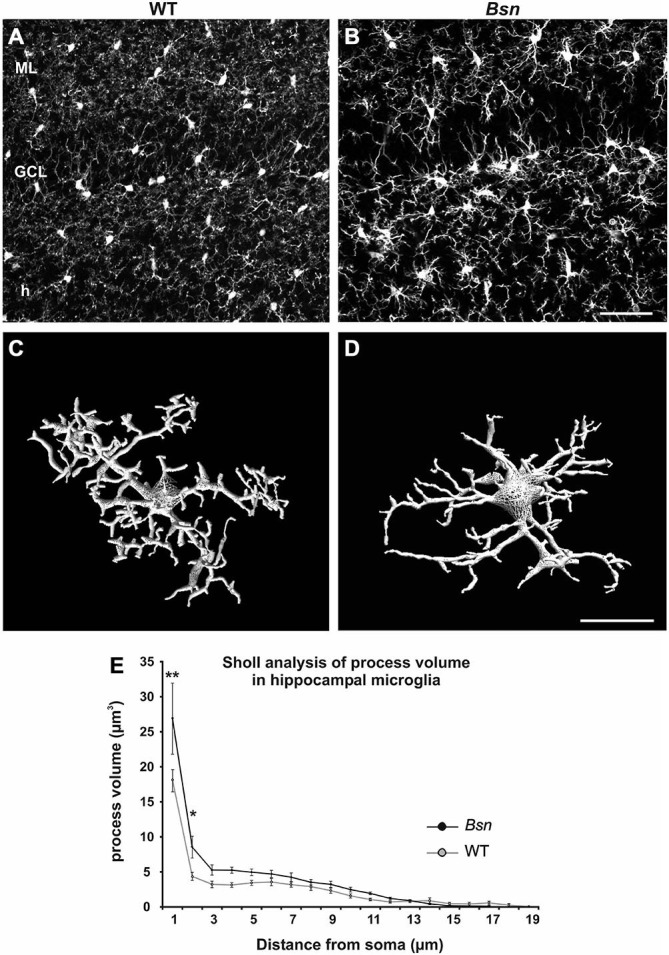
**Hippocampal microglia from *Bsn-*mutant mice show altered morphology.** Comparative Iba1 staining in hippocampi from WT and *Bsn-*mutant **(A)** In WT mice, microglia are evenly distributed throughout the layers of the DG and are characterized by a relatively small, round soma with thin, finely-branched processes. **(B)** A similar distribution was observed in the Bsn DG, however, microglia exhibited larger somata and thicker processes. **(C,D)** 3D-reconstruction of Iba-1-labeled microglia in the hilus depicts characteristic morphological changes at the cellular level. **(C)** Microglia from WT animals displayed ramified processes that were radially distributed around the cell soma. **(D)** Microglia from *Bsn*-mutants showed enlarged cell bodies and thicker processes close to the soma. **(E)** Sholl analysis confirmed the latter finding. Data are expressed as mean ± SEM. *p < 0.05; **p < 0.01. Scale bars: **A,B** = 50 μm; **C,D** = 2 μm. Abbreviations: ML, molecular layer; GCL, granule cell layer; h, hilus.

Increasing evidence suggests that microglial cells are critically involved in the regulation of neuronal plasticity, both during development (Paolicelli et al., 2011) and in the mature brain (Jones and Lynch, 2015). Since these cells are also emerging as important contributors to the pathophysiology underlying epileptic seizure activity (e.g., Eyo et al., 2014), WT and *Bsn*-mutant hippocampal sections were labeled with the microglia-specific antibody Iba-1 to determine whether neuronal hyperactivity influences microglial morphology in the DG. In the WT DG, microglia exhibited a typical ramified morphology (reviewed in Olah et al., 2011) and were evenly distributed across neuropil and cellular layers (Figure 6A). In the mutant DG, the microglia showed a similar distribution to the WT DG, but the individual cells showed stronger Iba-1-IR and appeared to have thicker processes (Figure 6B). To further analyze the potential morphological changes in mutant microglia, individual Iba1+ cells in the hilus underwent 3D reconstruction using NeuronStudio software (Wearne et al., 2005). The length, volume and surface area of microglial processes, as well as the number of branch points, were then determined using an automated Sholl analysis. In reconstructed microglia from WT animals, processes were ramified and radially distributed around the cell soma (Figure 6C), whereas microglia from *Bsn*-mutants showed enlarged cell bodies and thicker processes close to the soma. (Figure 6D).

Using a 2-way ANOVA to compare Sholl data between WT and *Bsn*-mutants, it was found that the Bassoon mutant genotype had a main significant effect on process volume (*F*_(1,328)_ = 8.882; *p* = 0.003), which was increased in comparison to WT microglia. There was also a significant interaction between “genotype” and “process distance from soma” (*F*_(16,328)_ = 1.695; *p* = 0.046). The strongest effects were evident on processes most proximal to the cell body (WT: 18.14 ± 5.07 μm^2^ vs. *Bsn*: 26.87 ± 1.59 μm^2^, *p* < 0.001; Figure 6E). The surface area of processes also differed significantly between genotypes (*F*_(1,328)_ = 7.414; *p* = 0.007); however, there was no significant interaction between “genotype” and “process distance from soma” (*F*_(16,328)_ = 0.672; *p* = 0.821). The Bassoon mutant genotype did not have any significant effects on microglial process length or the number of branch points. The results of the Sholl analysis therefore indicate that the Bassoon mutation leads to a selective but significant change in microglial morphology, suggesting that these cells are reactive and in turn, may have functional effects on the local neuronal environment.

### Mossy Fiber-CA3 Synapses Show Changes at the Ultrastructural Level

**Figure 7:**
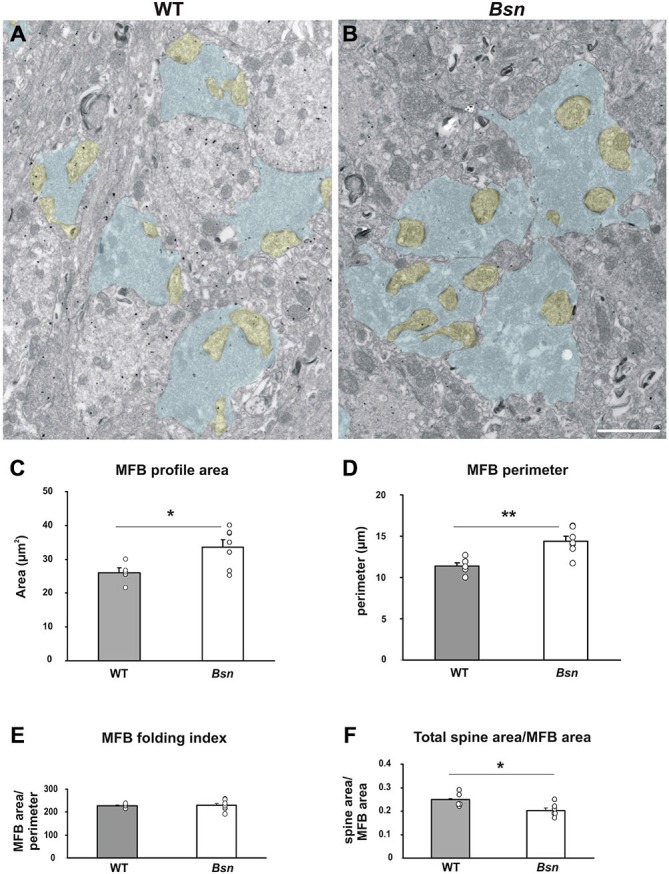
**Mossy fiber boutons from Bassoon mutants are enlarged compared to those from WT animals.** Representative electron micrographs of stratum lucidum in **(A)** WT mice vs. **(B)** *Bsn*-mutants. Selected MFB profiles are shaded in blue, while the corresponding spine profiles are shaded yellow. Note the larger MFB profiles in **B** (compared to **A**). Morphometric analysis of MFB profiles confirmed a significant increase in the area **(C)** and perimeter **(D)** of *Bsn-*mutant MFBs compared to WT mice, while the folding index (area/perimeter) remain unchanged **(E)**. **(F)** Graph showing that the increase in MFB area is not accompanied by an increase in the area of the complex spines contacting the corresponding bouton. Bar graphs represent mean values ± SEM (error bars), circles show individual mean values for each animal analyzed. *p < 0.05, **p < 0.005 (Student’s *t*-test). Scale bar: **(A,B)**: 2 μm. Abbreviations: MFB, mossy fiber bouton.

The findings described thus far point to changes in synaptic plasticity regulation at the cellular level; therefore, the next step was to identify potential alterations at the ultrastructural level which could serve as a structural substrate for the observed changes. Since the MF-CA3 synapse has previously been shown to undergo morphological changes in response to chemically-induced synaptic activity (Zhao et al., 2012), we wanted to determine whether similar synaptic changes in MFB ultrastructure were observed in the *Bsn*-mutant, which is also characterized by increased network activity. Using ultrathin sections, the respective areas of MFB profiles and their post-synaptic partners (CA3 complex spines) were quantified and compared between genotypes. MFB profiles in the SL of WT hippocampi exhibited typical structural characteristics such as a large surface area, multiple contacts with CA3 complex spines and a high number of synaptic vesicles (Figure 7A). In comparison, qualitative assessment of *Bsn*-mutant MFBs showed a similar morphology, but appeared larger than their WT counterparts (Figure 7B). This observation was confirmed by quantitative analysis, where the mean profile area of *Bsn* MFBs was significantly higher than that of WT MFBs (WT: 2.6 ± 0.15 μm^2^ vs. *Bsn*: 3.4 ± 0.22 μm^2^, *p* < 0.05; Figure 7C). Accordingly, *Bsn* MFB profiles also had a significantly higher perimeter than WT MFBs (WT: 11.4 ± 0.33 μm vs. *Bsn*: 14.4 ± 0.59 μm, *p* < 0.005; Figure 7D). To determine whether *Bsn* MFBs harbored a more convoluted structure than WT MFBs, MFB area was divided by MFB perimeter to determine the so-called folding index (Zhao et al., 2012). Here, no significant differences were observed between genotypes (Figure 7E), suggesting that *Bsn* MFBs do not acquire a more complex structure in the face of chronic changes to neuronal activity. Regarding mean spine area and number per MFB, no differences between genotypes were detected in either case; however, when mean total spine area was divided by mean total MFB area, WT animals showed a significantly higher ratio (WT: 0.250 ± 0.003; *Bsn*: 0.203 ± 0.010, *p* < 0.05; Figure 7F). In summary, these data suggest that while *Bsn* MFBs are enlarged compared to WT MFBs, they do not take on a more complex structure or increase the number of their post-synaptic elements. Alternatively, this structural change might represent the development of compensatory mechanisms such as enhanced endocytosis, which remains to be addressed in additional studies.

### BDNF-Containing Dense Core Vesicles Accumulate at the Presynaptic Membrane of Mossy Fiber Synapses

**Figure 8:**
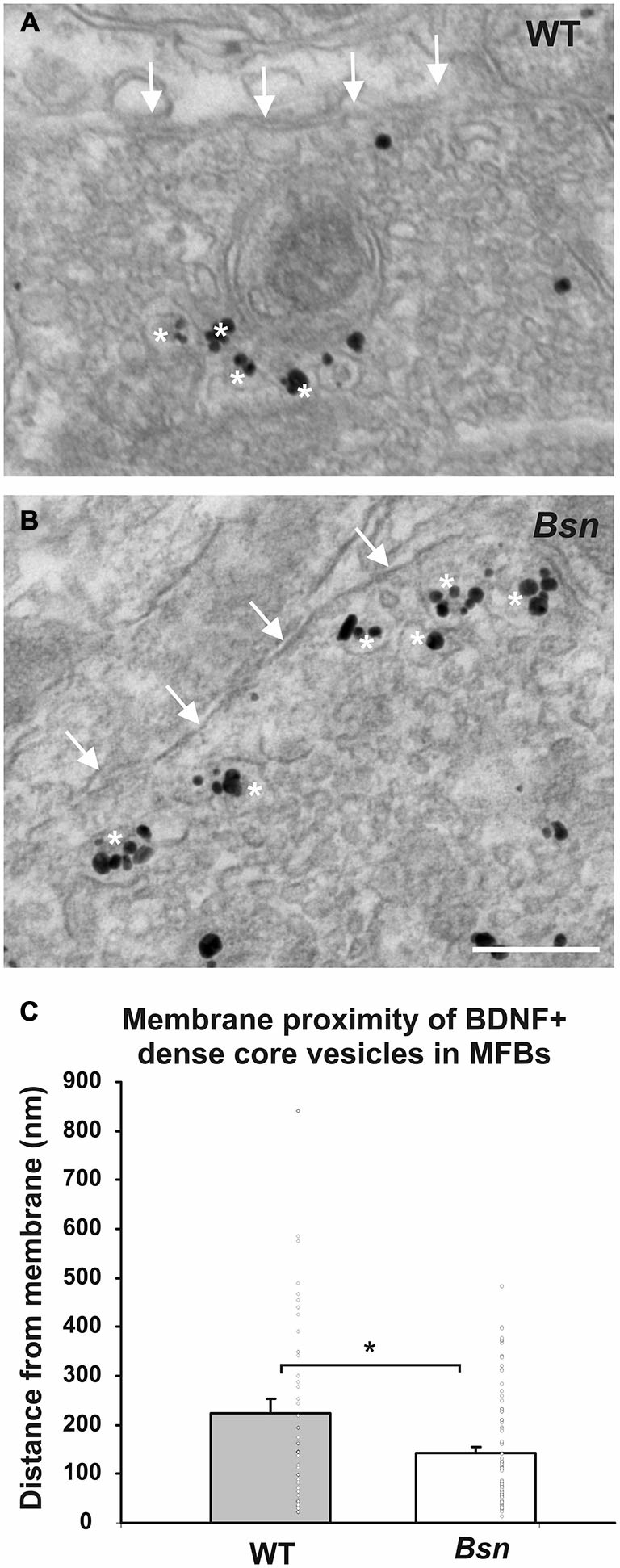
**BDNF-positive DCVs accumulate at the synaptic membrane in MFBs from *Bsn*-mutant mice.** Immunogold-labeled MFB showing the relative position of BDNF+ DCVs (white asterisks) and the MFB terminal membrane (arrows) in **(A)** WT mice and **(B)** *Bsn-*mutants. **(C)** Quantification of vesicle distance from the membrane revealed that BDNF+ DCVs accumulate closer to the synaptic membrane in *Bsn-*mutants compared to WT mice. Data are shown as mean ± SEM, circles represent single data points. *p < 0.05. Scale bar = 200 nm. Abbreviations: MFB, mossy fiber bouton.

The finding that *Bsn*-mutants MFBs have larger surface areas and perimeters also suggests that they have an increased storage capacity for synaptic vesicles. We previously reported that *Bsn*-mutant MFBs have a ~two-fold increase in density of BDNF+ DCVs, as determined by immunogold labeling (Dieni et al., 2012). Whether this corresponds to increased release of BDNF has not yet been determined. To begin to address this question, the degree of accumulation of BDNF+ DCVs at the synaptic membrane of the MFB was quantified. By measuring the most direct distance of individual BDNF-labeled DCVs from the synaptic membrane, it was found that BDNF+ vesicles in WT MFBs had a mean distance of 240 ± 29 nm from the synaptic membrane (Figures 8A,C), but were situated closer to the membrane in *Bsn* MFBs, with a mean distance of 140 ± 13 nm (Figures 8B,C; *p* < 0.05). These results therefore suggest that there is at least an increased accumulation of BDNF+ DCVs near release sites in *Bsn*-mutant MFBs.

## Discussion

The present study was undertaken to evaluate whether the altered synaptic activity in the *Bsn*-mutant affects the ultrastructure of a defined synapse type (MF synapses in the hippocampus), as well as various molecules directly or indirectly involved in synaptic transmission. Our detailed structural and immunohistochemical analyses revealed that the increased BDNF levels characterizing the *Bsn*-mutant hippocampus are associated with: (i) modified neuropeptide distribution; (ii) perturbed expression of selected markers of synaptic activation or plasticity; (iii) subtle changes to microglial structure; and (iv) morphological alterations at the MF-CA3 synapse.

### Altered Neuropeptide Distribution Accompanies Increased Neurotransmission Within the MF Pathway of the *Bsn*-Mutant

As previously discussed (Dieni et al., 2012), the observed increase in BDNF levels in the *Bsn*-mutant is in line with earlier studies in the rat, in which chemically-induced seizure activity led to an increase in presynaptic BDNF staining (Vezzani et al., 1999; Scharfman et al., 2002; Danzer and McNamara, 2004). The present study now extends these data by demonstrating that increased neuronal activity in the *Bsn*-mutant is also associated with changes in the immunohistochemical distribution of the neuropeptides Met-enk and CCK. These findings are corroborated by earlier limbic seizure studies in rodents, which reported a dramatic upregulation of Enkephalin-IR (Gall et al., 1988; Gall, 1988), and a selective downregulation of CCK-IR (Gall, 1988; Arabadzisz et al., 2005) in MFs.

The similar pattern of upregulation observed for both BDNF and met-enk in the *Bsn*-mutant points to an increased number of MFBs that contain both peptides. Moreover, since Met-enk-positive granule cells invariably co-express BDNF (Dieni et al., 2012), it is likely that both peptides are trafficked to the same MF terminals. However, whether this suggests that BDNF and Met-enk respond to the same stimuli required for transcription and release remains to be tested. The functional consequences of elevated Met-enk synthesis and (presumably) release might pertain to overall excitability in the *Bsn* hippocampus, since enkephalin was previously shown to hyperpolarize hippocampal interneurons, which in turn led to disinhibition of both pyramidal cells and inhibitory interneurons (Madison and Nicoll, 1988).

There is little evidence to suggest that BDNF and CCK are governed by similar regulatory mechanisms in the hippocampus, since CCK is down-regulated regardless of whether BDNF is endogenously overexpressed, or genetically ablated. The latter is supported by the observation that CCK-IR was almost completely down-regulated in forebrain-specific CaMK-BDNF^KO^ mutants (Vigers et al., 2012). However, the mechanisms leading to downregulation of CCK remain obscure. In any case, the selective loss of CCK could have implications for hippocampal plasticity (Li et al., 2014), particularly since CCK has been shown to rescue morphine-induced LTP deficits in the hippocampus (Wen et al., 2014).

Therefore, despite the fact that BDNF, met-enk and CCK are all small neuropeptides derived from a larger precursor, it is likely that they are governed by different regulatory mechanisms, and that the normal relative proportions of neuropeptides are perturbed in *Bsn*-mutants. Furthermore, the observed change in neuropeptide distribution with the MF system may contribute to ongoing changes in seizure susceptibility, as previously hypothesized (Gall, 1988).

### Marked Loss of Calbindin-Immunoreactivity in the *Bsn*-Mutant Hippocampus

Under normal conditions, Calbindin-D28K is ubiquitously expressed in dentate granule cells, where it acts as a calcium buffer and serves to maintain calcium homeostasis (Mattson et al., 1991). In line with the present observation of CB loss in *Bsn-*mutant granule cell bodies, dendrites and axons, previous work in animal models of chemically-induced epilepsy (Bouilleret et al., 2000; Gary et al., 2000) as well as in human TLE (Maglóczky et al., 1997; Nägerl et al., 2000; Selke et al., 2006), also demonstrated a loss of CB-IR in this cell population. While several reports suggest that Calbindin does not necessarily protect granule cells from kainate-induced excitotoxicity (Gary et al., 2000), the loss of CB-IR in human dentate granule cells has been hypothesized to result in hyperexcitability of the DG (Maglóczky et al., 1997). Moreover, given that hippocampal calcium homeostasis is known to be disrupted in acquired forms of epilepsy (Delorenzo et al., 2005), and intracellular calcium signaling is critical to the maintenance of neuronal function (Mattson et al., 2000), one likely outcome of CB loss in *Bsn*-mutant dentate granule cells is an overall negative effect on neuronal viability and synaptic plasticity. In support of this, lower CB-IR was found to correspond to poorer memory performance in aged mice (Soontornniyomkij et al., 2012), while an absence of CB in the hippocampus led to a disruption of LTP maintenance in both the DG and CA1 (Westerink et al., 2012). Taken together, the loss of CB-IR in the *Bsn*-mutant DG is likely to have a detrimental effect not only on cytosolic calcium homeostasis *per se*, but also on calcium-related events occurring at the pre- and post-synaptic levels of the granule cell.

### Differential Changes in Synaptic Activation Markers

Given that presynaptic BDNF levels are increased in the *Bsn*-mutants, it was important to determine whether this was associated with an upregulation of other proteins normally influenced by elevated synaptic activity. We found opposing changes in the markers Arc and synaptopodin, which may have implications for overall synaptic function.

The chronic changes in neuronal excitability in the *Bsn* hippocampus were associated with a dramatic downregulation of Arc protein expression in the GCL, with little evidence of dendritic staining and only scattered Arc-labeled nuclei. Studies investigating the acute effects of chemical- or lesion-induced seizure on Arc expression have essentially demonstrated an upregulation of the mRNA or protein. For example, hippocampal Arc mRNA was upregulated in acute response to KA-induced seizure (Plath et al., 2006), while Arc protein expression during kindling was observed in the DG (Szyndler et al., 2013). Less information is available about the long-term effects of seizures on Arc expression; however, in support of our findings, one study investigating the long-term neuronal adaptations to electroconvulsive seizures found after more than 24 h post-seizure reduced expression levels of IEGs including Arc (Calais et al., 2013).

The reasons underlying the observed Arc downregulation in *Bsn*-mutant granule cells are not clear; however, the functional implications for the loss of local Arc protein synthesis are likely to relate to multiple forms of synaptic plasticity, since, for example, LTP in the rat DG was shown to correspond to Arc protein expression in spines and dendrites (Rodriguez et al., 2005), and a loss of Arc expression led to increased AMPAR function and abolished homeostatic scaling of AMPA receptors (Shepherd et al., 2006). Indeed, LTP is impaired at CA1 synapses (Sgobio et al., 2010) and striatal spiny neurons (Ghiglieri et al., 2009) in *Bsn*-mutant mice.

Previous studies have shown that synaptopodin expression is altered in response to kainic acid injection (Roth et al., 2001), and is upregulated in parallel with other cytoskeletal proteins following induction of LTP *in vivo* (Fukazawa et al., 2003). Thus, synpo-IR can serve as a useful marker of potential changes in post-synaptic activity status. Despite the dramatic reduction in Arc expression, synpo-IR was markedly increased in the *Bsn*-mutant DG, with stronger fluorescence intensity and qualitatively larger puncta. These unexpected findings indicate that the altered neuronal activity in the *Bsn* hippocampus promotes an increase in synpo expression, which may lead to spine enlargement (Okubo-Suzuki et al., 2008). This effect could also be related to increased presynaptic stimulation of dendritic spines in the MML and OML by entorhinal afferents. It should be noted, however, that no changes in spine number or size were observed at the MF/CA3 synapse at the ultrastructural level.

### Morphological Changes to Microglia in Chronic Neuronal Hyperactivity

Microglial cells are fine sensors of altered neuronal activity (Béchade et al., 2013). Indeed, a typical feature of microglia activation in response to increased neuronal activity is an up-regulation of Iba1 (Avignone et al., 2008; Shapiro et al., 2008; Wirenfeldt et al., 2009; Cepeda et al., 2015). Furthermore, detailed morphological analyses revealed that microglia exhibit larger somata and thicker proximal processes in response to general convulsion activity (Avignone et al., 2008). In line with these findings, we also detected an increase in the volume of the most proximal microglial processes in the current model of chronic neuronal hyperactivity. Thus, the altered neuronal activity resulting from the *Bsn* protein mutation leads to a selective but significant change in microglial morphology. Given the increasing evidence that changes in microglial structure closely correspond to functional changes, our observations suggest that microglia functionally adapt to the network changes that characterize the *Bsn*-mutant.

### Contribution of Reelin to Network Excitability

Reelin has been shown to modulate NMDA receptor activity (Chen et al., 2005) and regulate neurotransmitter release in the hippocampus (Hellwig et al., 2011). Moreover, Reelin secreted by GABAergic interneurons reportedly modulates NMDA receptor homeostasis (Campo et al., 2009). Although the GABAergic phenotype of hippocampal interneurons is in part modulated by BDNF (Subburaju and Benes, 2012), the dramatic increase in presynaptic BDNF protein levels in *Bsn*-mutant mice did not appear to influence the Reelin-positive subpopulation of GABAergic interneurons in this setting.

Reelin has been linked to the pathophysiology underlying TLE, which represents a state of hyperexcitability in temporal lobe structures (Haas et al., 2002). Kainate-induced GCD, a hallmark of TLE (Houser, 1990), is associated with fewer Reelin+ interneurons in the hilus (Chai et al., 2014). The lack of change in Reelin+ hilar interneurons in *Bsn*-mutant mice thus corresponds to the normal GCL architecture, as previously reported in these mutants (Heyden et al., 2011).

### Altered Synaptic Morphology following Chronic Changes to Neuronal Activity

Ultrastructural analysis of SL revealed that *Bsn*-mutant MFBs are enlarged compared to WT MFBs, which could partially contribute to the overall increase in hippocampal volume in these mutants (Heyden et al., 2011). However, since this was neither associated with a more complex membrane structure, nor an increase in the number of post-synaptic elements, it could be surmised that similar structural plasticity as described recently after the induction of chemical LTP (see Zhao et al., 2012) is not occurring at the MF-CA3 synapse in *Bsn*-mutants. An electrophysiological study performed during postnatal development demonstrated that functional maturation of MF–CA3 synapses in *Bsn*-mutant mice is an impaired process which, however, normalizes by P21 (Lanore et al., 2010). A potential structural correlate for this was an increase in the number of active zones at P7 (but not at later postnatal time points). Whether this perturbed developmental process accounts for the altered MFB structure in adult *Bsn*-mutants remains to be determined.

We previously observed an increase in the number and density of BDNF+ DCVs in *Bsn*-mutant MFBs (Dieni et al., 2012), where qualitative observations were suggestive of an accumulation of labeled DCVs in proximity to the synaptic membrane. Measurement of vesicle-to-membrane distance in the present study confirmed this, with a significantly higher number of BDNF+ DCVs located closer to the MFB membrane in *Bsn* vs. WT mice. This finding suggests that either more BDNF is poised for release from *Bsn* MFBs, or that the release mechanisms for DCVs are impaired in the absence of functional *Bsn* protein, leading to an accumulation of BDNF-containing vesicles.

## Conclusion

In summary, this study has used the *Bsn*-mutant mouse line to show that a chronic imbalance in neuronal network activity is associated with neurochemical and ultrastructural changes that are likely to directly or indirectly affect synaptic function. In addition, despite the dramatic increase in BDNF levels in *Bsn*-mutants, our immunohistochemical and ultrastructural data instead point towards an accumulation of the neurotrophin in MFBs, which might result from the lack of a proper release stimulus due to the altered physiological environment in *Bsn*-mutants. Whether the findings reported here are a direct result of the *Bsn* mutation and in particular, whether Bsn affects the release mechanism of BDNF-containing DCVs, should be the focus of future studies.

## Author Contributions

Designed the study: SD, SH; Performed the experiments: SD, SN; Analyzed the data: SD, SN, MS, SH; Interpreted the results: SD, SN, MF, SH; Edited the manuscript: MS, MF; Wrote the manuscript: SD, SH.

## Funding

This study was supported by the Hertie Foundation (MF), GE Healthcare Buchler GmbH & Co. KG (SH) and the Medical Faculty of the University of Freiburg (SH).

## Conflict of Interest Statement

The authors declare that the research was conducted in the absence of any commercial or financial relationships that could be construed as a potential conflict of interest.

## Footnotes

^1^http://www.spss.com
